# Integrated analysis of patients with KEAP1/NFE2L2/CUL3 mutations in lung adenocarcinomas

**DOI:** 10.1002/cam4.4338

**Published:** 2021-10-06

**Authors:** Xing Jin, Yuansheng Zheng, Zhencong Chen, Fei Wang, Guoshu Bi, Ming Li, Jiaqi Liang, Qihai Sui, Yunyi Bian, Zhengyang Hu, Yulei Qiao, Songtao Xu

**Affiliations:** ^1^ Department of Thoracic Surgery Zhongshan Hospital Fudan University Shanghai China; ^2^ Taizhou People’s Hospital Taizhou Jiangsu China; ^3^ Department of Thoracic Surgery Zhongshan Hospital Fudan University (Xiamen Branch) Xiamen Fujian China

**Keywords:** cullin 3 (CUL3), kelch‐like ECH‐associated protein 1 (KEAP1), lung adenocarcinoma (LUAD), mutation, nuclear factor erythroid 2‐like 2 (NFE2L2)

## Abstract

**Objectives:**

To explore the clinical features, molecular characteristics, and immune landscape of lung adenocarcinoma patients with KEAP1/NFE2L2/CUL3 mutations.

**Methods:**

The multi‐omics data from the GDC‐TCGA LUAD project of The Cancer Genome Atlas (TCGA) database were downloaded from the Xena browser. The estimate of the immune infiltration was implemented by using the GSVA analysis and CIBERSORT. The status of KEAP1/NFE2L2/CUL3 mutation in 50 LUAD samples of our department was detected by using Sanger sequencing, following the relative expression level of differentially expressed genes (DEGs), miRNAs (DEmiRNAs), and lncRNAs (DElncRNAs) was validated by IHC and real‐time quantitative polymerase chain reaction (RT‐qPCR).

**Results:**

The Kaplan–Meier and multivariable Cox regression analyses demonstrated that KEAP1/NFE2L2/CUL3 mutations had independent prognostic value for OS and PFS in LUAD patients. The differential analysis detected 207 upregulated genes (like GSR/UGT1A6) and 447 downregulated genes (such as PIGR). GO, KEGG, and GSEA analyses demonstrated that DEGs were enriched in glutamate metabolism and the immune response. The constructed ceRNA network shows the linkage of differential lncRNAs and mRNAs. Three hundred and nine somatic mutations were detected, alterations in immune infiltration DNA methylations and stemness scores were also founded between the two groups. Eight mutated LUAD patients were detected by Sanger DNA sequencing in 50 surgical patients. GSR and UGT1A6 were validated to express higher in the Mut group, whereas the expression of PIGR was restrained. Furthermore, the IHC staining conducted on paraffin‐embedded tissue emphasizes the consistency of our result.

**Conclusion:**

This research implemented the comprehensive analysis of KEAP1/NFE2L2/CUL3 somatic mutations in the LUAD patients. Compared with the wild type of LUAD patients, the Mut group shows a large difference in clinical features, RNA sequence, DNA methylation, and immune infiltrations, indicating complex mechanism oncogenesis and also reveals potential therapeutic targets.

## Graphical abstract

For the first time, an integrated multi‐mics analysis of KEAP1/NFE2L2/CUL3 mutated LUAD patients was performed. Based on several independent database, we validated the clinical value of KEAP1/NFE2L2/CUL3 in univiarate and multiviarate analyses. Our research shed light on the significance of potential therapeutical target via bioinformatics analyses and experimental verification.

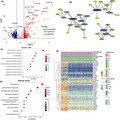

## 1 INTRODUCTION

According to the newest data, lung cancer is the second most common cancer diagnosis and the first main reason for cancer death.^1^ The survival of patients with lung cancer at 5 years after diagnosis is only 10%–20%. Lung adenocarcinoma accounts for most lung cancer cases, and its incidence has been increasing year by year.^2^, ^3^ Although targeted therapy and immunotherapy have led to dramatic changes in lung cancer treatment, the resistance to the therapies and intratumor heterogeneity has become a new challenge. It is imperative to exploit the new potential target of molecularly targeted therapies.^4^

Kelch‐like ECH‐associated protein 1 (KEAP1) mutation is one of the most common lung cancer mutations, and its mutant frequency has been over 20% in lung adenocarcinoma (LUAD). KEAP1 mutation often disrupts the interaction of KEAP1/NFE2L2/CUL3, and its disability leads to the promotion of tumor genesis through the abnormal activation of NFE2L2.^5^, ^6^ Early in 2014, a study of the TCGA Research Network revealed that KEAP1/NFE2L2/CUL3 somatic alterations were components of one of the key pathways in LUAD. However, the patients with KEAP1/NFE2L2/CUL3 mutations clinical characteristics remain unclear. The effect of these mutations and the pathways’ mechanisms are still under investigation. Despite many drugs targeting the key genes (EGFR, KRAS) mutation developed and applied into the first‐line usage,^7^, ^8^, ^9^, ^10^, ^11^ the agents targeted to the KEAP1 gene are still not available to date.

Our study, based on the multi‐omics data from the TCGA database, integrated the clinical data and expression profiles, comprehensively analyzed the differences in clinical features, somatic nucleotide variations, gene expression, transcriptome, and tumor immune microenvironment between the KEAP1/NFE2L2/CUL3 pathway mutant and the wild‐type patients in LUAD. The present study aims to increase the understanding of KEAP1/NFE2L2/CUL3 mutations in LUAD and shed light on new drugs targeting this pathway.

## 2 MATERIALS AND METHODS

### 2.1 Data acquisition

The gene expression data (log(FPKM+1)) (reads per kilobase per million) of 585 LUAD patients (493 LUAD tissues were used) and corresponding clinical information of the Cancer Genome Atlas (TCGA) were downloaded from the UCSC Xena browser (GDC hub: https://gdc.xenahubs.net). We removed patients whose survival time, new event time, or vital status were indefinite. The copy number variation and DNA methylation (Methylation 450k) data of TCGA were normalized and downloaded by the UCSC Xena browser. The miRNA expression data and somatic mutation (VarScan MAF files) were downloaded from TCGA (https://tcga‐data.nci.nih.gov/tcga/findArchives.htm). All the data were matched with their group information during further analysis.

### 2.2 **Clinical** data analysis

The OS (Overall Survival) and PFS (Progression‐Free Survival) analyses were performed using the R package survival analysis. Afterward, univariate Cox regression and multivariate Cox regression analyses were conducted by survival package. The construction of the nomogram plot was based on the results of the Cox analysis. Besides, the Concordance index (C‐index) was used to determine the discrimination ability of the nomogram. The calibration curve of the nomogram was plotted to observe the nomogram prediction probabilities.

### 2.3 Somatic mutations and copy number variants

Mutation Annotation Format (MAF) files that reserve information about somatic mutations was summarized, analyzed, annotated, and visualized using the maftools Bioconductor package.^12^ We also compared copy number variations (CNVs) between the two groups. The different SNPs between the two groups were detected using the mafCompare function in the maftools package, which performs a Fisher test on all SNPs, and we set the *p* value of <0.01 as the screening threshold.

### 2.4 **D**ifferentially expression analysis

The mRNAs and lncRNAs were annotated by using the Genecode database (https://www.gencodegenes.org/, version: Release 22 [GRCh38.p2]) ^13^; the miRNAs were annotated by using the R package named “miRBaseVersions.db.” According to the gencodes annotation files, all 15328 lncRNAs were extracted from the mRNA expression matrix. Differentially expressed mRNAs, miRNAs, and lncRNAs (DEmRNA, miRNAs, and lncRNAs) were identified in Mut and Wild groups using package limma.^14^ Specifically, expression data were input and underwent lmFit and eBayes functions in the R limma package. Then we set the cutoff criteria of screening differentially expressed genes as adjust. *p* value <0.05 and logFC(log(Fold Change)) >0.5.

### 2.5 Functional enrichment analyses

Gene Ontology (GO) terms and Kyoto Encyclopedia of Genes and Genomes (KEGG) pathways enrichment analyses performed based on the GO database (http://www.geneontology.org/) and the KEGG database (http://www.genome.jp/kegg/). The R package “ClusterProfiler” was used to distinguish the differentially expressed pathways, with p‐values calculated using right‐sided hypergeometric, and the package “enrichplot” was used for visualization.

### 2.6 PPI network and ceRNA construction

PPI networks were established using STRING,^15^ v11.0 by uploading the DEG list, and the isolated nodes were deleted. An exported .cys file format from STRING has then conducted the polishment by Cytoscape. Based on the ceRNA hypothesis, a ceRNA network's construction was built by the “GDCRNATools”^16^ R package, the gdcCEanalysis function identified ceRNAs by some databases of miRNA–lncRNA interactions like starBase, we set the thereshold as hyperPvalue as 0.01 and CorPvalue as 0.01. Both networks were visualized and polished by Cytoscape^17^ software (Version 3.8.3).

### 2.7 Immune infiltration analysis

To construct a Geneset of microenvironment genes to divide immune cell subsets, we accepted the investigation of Bindea et al.^18^, ^19^, ^20^ It incorporated 585 genes to 24 immune cell subcollections from intrinsic and adaptive immunity. The 24 immune‐related cells contain dendritic cells (DCs), immature DCs, activated DCs (aDCs), macrophages, mast cells, neutrophils, eosinophils, natural killer (NK) cells, NK CD56dim cells, NK CD56bright cells, T cells, and CD8 T cells, as well as Tγδ, T helper, Tcm, Tem, Th1, Th2, Th17, Tfh, Tgd, Treg cells, B cells, and cytotoxic cells. The expression values of immune cells were calculated from protein‐coding mRNA’s log(FPKM+1) via R “GSVA” package^19^ with the following parameters: method = “gsva,” mx.diff = “TRUE,” and kcdf = “Gaussian.” We used the ImmuCellAI^21^ and CIBERSORT (https://cibersort.stanford.edu/), EPIC, and QUANTISEQ^22^ algorithm to predict the immune cell proportions and the immune infiltration score.

### 2.8 Differential analysis of DNA methylation and Stemness index

Differentially methylation positions (DMP) were identified by Fisher's exact test using the R package “ChAMP”.^23^ The GSEA (Gene Set enrichment analysis) (https://www.gsea‐msigdb.org/gsea/index.jsp) of DMR, and DMP was conducted through the “champ.gsea”^24^, ^25^, ^26^, ^27^ function in ChAMP. Stemness indices were collected from a Malta study,^28^ and we applied mRNAsi and mDNAsi to identify the stemness based on mRNA and DNA methylation expression.

### 2.9 RNA isolation from patients’ tumor tissue and real‐time PCR

LUAD tumor tissues of 50 patients were obtained from the Department of Thoracic Surgery, Zhongshan Hospital, Fudan University, Shanghai, China, who had received surgery from November 2020 to May 2021.

Total RNA from the patients’ samples was extracted using TRIzol reagent (TIANGEN Biotech, Beijing, China). The cDNA synthesis was performed using the PrimeScript^TM^ RT Master Mix (Yeasen, Shanghai, China). Real‐time PCR was conducted with the SYBR‐Green kit (Yeasen) to detect the mRNA expression levels of core prognostic genes. The gene and lncRNA primer were listed in Table S1.

miRNA preparation and detection procedures were performed as previously reported.^29^ The total miRNAs were extracted by miRcute miRNA Isolation Kit (TIANGEN), and the miRNA First‐Strand cDNA Synthesis Kit (TIANGEN) was used to synthesize miRNA cDNA according to the manufacturer's instructions. miRcute miRNA qPCR Detection Kit (SYBR Green) (TIANGEN) was used with the following PCR parameters, 1 cycle of 2 min at 94°C, 40 cycles of 20 s at 94°C, and 40 cycles of 34 s at 60°C using a QuantStudio™ 5 Real‐Time PCR Systems. (Thermo Fisher Scientific, Inc.). miRNA primers were obtained from TIANGEN.

### 2.10 Sanger sequencing

Sanger sequencing was performed as previously reported.^30^ First, cDNA was amplified using 2 × HotStart Taq PCR MasterMix (TIANGEN). Then the PCR products were sequenced by Sangon Biotech (Shanghai, China). Sequencing results were compared with corresponding entries in the National Centre for Biotechnology Information (NCBI) Nucleotide Database (http://www.ncbi.nlm.nih.gov/nuccore/, NFE2L2: NM_006164.5, KEAP1: NM_203500.2, CUL3: NM_003590.5). Reduplicated experiments further confirmed all of the mutations detected. Single nucleotide polymorphism (SNP) information was obtained from the NCBI dbSNP database (http://www.ncbi.nlm.nih.gov/snp/).

### 2.11 IHC staining

Mut LUAD and paired Wild LUAD paraffin‐embedded tumor tissues of 18 patients were also obtained. Primary antibodies used in IHC, including GSR (ab134315, 1:200 for IHC), UGT1A6 (ab157476, 1:250 for IHC), PIGR (ab275020, 1:200 for IHC), all antibodies were purchased from Abcam, Cambridge, UK. The procedure was constructed as previously reported.^31^ For quantification of IHC images, the ImageJ IHC Toolbox plugin was used in ImageJ software (NIH).

### 2.12 Statistical analysis

The whole statistical analysis was performed using R studio and R software (Version 4.0.4; R Foundation for Statistical Computing). The distribution of baseline characteristics between the Wild and Mut groups was analyzed in which categorical variables were compared by the chi‐square test and Fisher's exact test when appropriate. Continuous variables were compared by the use of the Students’ *t* test and Wilcoxon test. Survival analysis performed the log‐rank test and Cox regression. Multivariate Cox regression analyses were conducted to determine the independent prognostic factors related to overall survival using the “step()” function in R. The forestplot, nomogram, and other plots were performed using the regplot, ggplot2, and forestplot. Data screen, transformation, and visualization were performed using the “tidyverse” packages. All *p* values were two‐sided, and *p* < 0.05 indicated statistical significance.

## 3 RESULT

### 3.1 Clinical Features

**FIGURE 1:**
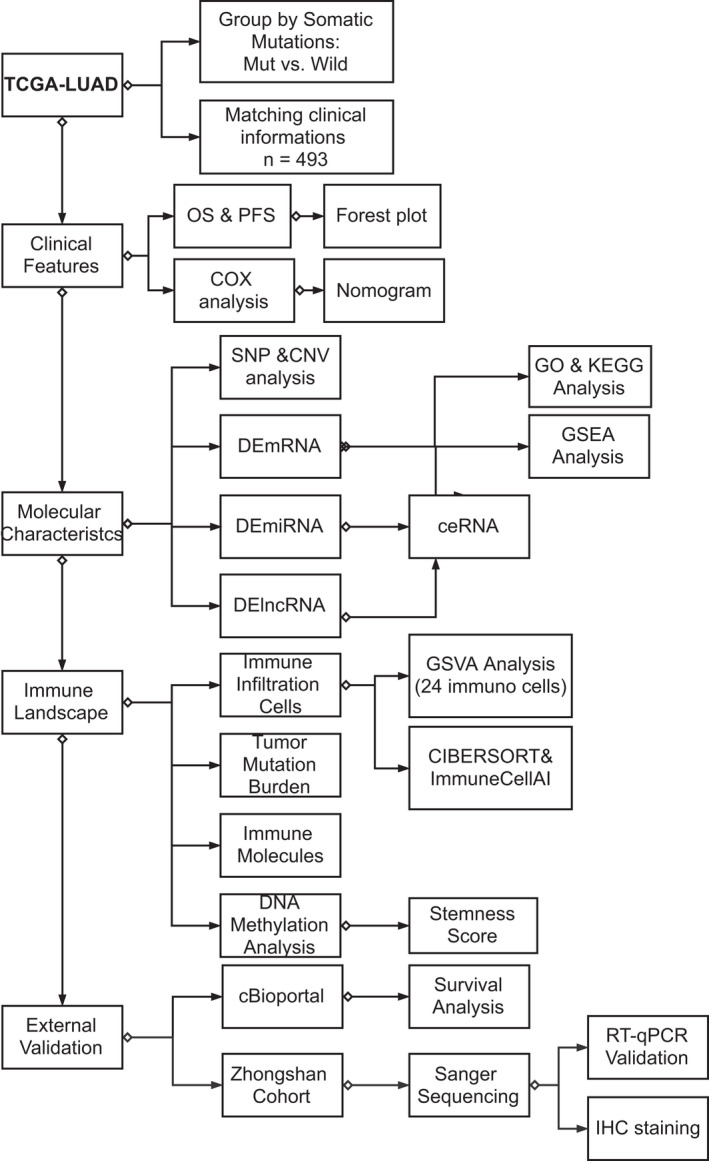
Flowchart diagram: a flow chart of the whole study design and analysis

The workflow of our research was shown in Figure 1. Table 1 shows the patients’ baseline characteristics (e.g., sex, age, race, and smoke group), summarized using counts and percentages. All 493 patients were separated into groups due to their mutation status. No significant divergence was observed in the two groups’ clinical characteristics, except for sex; more male patients were shown in the KEAP1/NFE2L2/CUL3 Mut group (male: 59.46% Mut, female: 40.54% Mut, *p = *0.002). Moreover, patients in the Smoke group are more likely to gain the KEAP1/NFE2L2/CUL3 mutations (smoke yes: 25.4% Mut, no/unknown: 18.3% Mut, *p = *0.087), although this difference was not statistically significant (Table 1). Furthermore, the tumor stage distribution with no significant difference indicated no association with the KEAP1/NFE2L2/CUL3 mutation status and clinical tumor stage or TNM stage.

**TABLE 1:** Baseline characteristics of the LUAD patients in two groups from the TCGA database

Characteristics	Mut (*n* = 111)	Wild (*n* = 382)	Overall (*n* = 493)	*p* value
Age (median [IQR])	65 [59, 72]	66 [59, 73]	66 [59, 72]	0.6142
Sex (%)				
Female	45 (40.54)	222 (58.12)	267 (54.16)	**0.0016**
Male	66 (59.46)	160 (41.88)	226 (45.84)	
Race (%)				
White	89 (80.18)	291 (76.18)	380 (77.08)	0.4364
Black	12 (10.81)	39 (10.21)	51 (10.34)	
Other	10 (9.01)	52 (13.61)	62 (12.58)	
Smoke_group (%)^a^				
No/Unknow	36 (33.33)	160 (43.13)	196 (40.92)	0.0872
Yes	72 (66.67)	211 (56.87)	283 (59.08)	
T (%)				
T1	36 (32.73)	130 (34.21)	166 (33.88)	0.9256
T2	59 (53.64)	205 (53.95)	264 (53.88)	
T3	11 (10.00)	31 (8.16)	42 (8.57)	
T4	4 (3.64)	14 (3.68)	18 (3.67)	
N (%)				
N0	72 (64.86)	245 (64.30)	317 (64.43)	0.9159
N1	21 (18.92)	73 (19.16)	94 (19.11)	
N2	17 (15.32)	52 (13.65)	69 (14.02)	
N3	0 (0.00)	2 (0.52)	2 (0.41)	
NX	1 (0.90)	9 (2.36)	10 (2.03)	
M (%)				
M0	67 (60.36)	261 (69.05)	328 (67.08)	**0.0147**
M1	11 (9.91)	13 (3.44)	24 (4.91)	
MX	33 (29.73)	104 (27.51)	137 (28.02)	
Stage group (%)^b^				
Early stage	80 (73.39)	302 (79.89)	382 (78.44)	0.1863
Later stage	29 (26.61)	76 (20.11)	105 (21.56)	
Resection site (%)				
Lower lobe	39 (35.14)	128 (33.51)	167 (33.87)	0.2533
Middle lobe	3 (2.70)	17 (4.45)	20 (4.06)	
Upper lobe	62 (55.86)	227 (59.42)	289 (58.62)	
Other site	7 (6.31)	10 (2.62)	17 (3.45)	
Radiotherapy (%)				
No/unknown	96 (86.49)	339 (88.74)	435 (88.24)	0.6296
YES	15 (13.51)	43 (11.26)	58 (11.76)	

^a^ Smoke_group: No/unknown: lifelong nonsmoker, reformed smoker for >15 years or smoke history not documented.

^b^ Early stage: stage I–II; later stage: III‐IV. TCGA, the Cancer Genome Atlas.

The bold values indicate the significant of *p* values.

**FIGURE 2:**
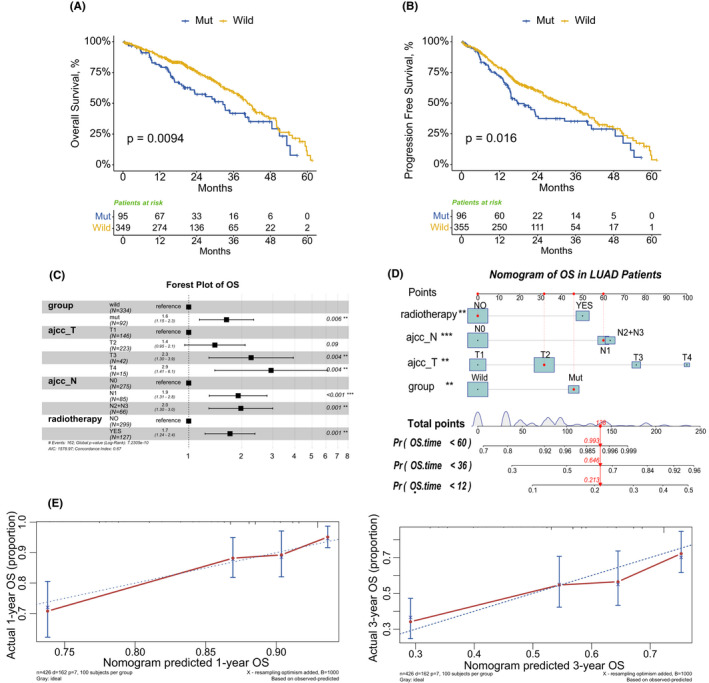
Survival analysis and nomogram. Survival curves and forest plots of overall survival and progression‐free survival in LUAD patients with or without the KEAP1/NFE2L2/CUL3 mutations. Kaplan–Meier survival curves show significant differences between the Mut and the Wild groups in overall survival (OS) (A) and progression‐free survival (B). The forest plots manifested that the KEAP1/NFE2L2/CUL3 mutations are a risk factor for LUAD patients in overall survival (OS) (C) Nomogram of the overall survival in LUAD patients (D). 1‐year and 3‐year internal calibration plots of the overall survival nomogram (E)

The log‐rank method was implemented to compare the OS (Overall survival) of LUAD patients in the Mut group and the Wild group (Figure 2A and Figure S1). Patients whose tumors carried KEAP1/NFE2L2/CUL3 mutations had significantly worse overall survival than their wild‐type counterparts (median survival time: Mut 32.5 months vs. Wild 40.5 months, *p* = 0.009). The progression‐free survival (PFS) analysis, which can better reflect tumor progression and predict clinical benefits, also showed an association between KEAP1/NFE2L2/CUL3 mutation and faster disease progression (median survival time: 17.7 months vs. 31.7 months, *p* = 0.016). Next, we undertook univariate and multivariate Cox regression analyses of the clinical characteristics listed in Table 1. The Cox hazard regression model results are shown in Table 2, which revealed that KEAP1/NFE2L2/CUL3 mutation is an independent prognostic factor for the patients’ prognosis (Univariate cox: HR 1.63 [1.14, 2.32], *p* = 0.007 and multivariate cox: HR 1.48 [1.08, 2.02], *p* = 0.014). Next, we constructed the nomogram to predict 1‐year and 3‐year OS based on the step‐wise multivariable cox model's result, including group, ajcc_T, ajcc_N, and radiotherapy (Figure 2D and Figure S2). The Nomogram's C‐index is 0.672; calibration plots showed the nomogram in the internal validation has a good prediction of the patients’ prognosis.

**TABLE 2:** Univariable and multivariable Cox regression analysis results of OS and PFS

Characteristics	OS (Over Survival)				PFS (Progression‐Free Survival)			
	Univariable		Multivariable		Univariable		Multivarible	
	HR (95% CI)	*p* value	HR (95% CI)	*p* value	HR (95% CI)	*p* value	HR (95% CI)	*p* value
Group: Wild								
Mut	1.53 (1.08–2.16)	0.018	1.63 [1.14, 2.32]	**0.007**	1.42 (1.04–1.94)	0.027	1.48 [1.08, 2.03]	**0.014**
Age	1 (0.98–1.02)	0.936	1.01 [1.00, 1.03]	0.138	1.01 (0.99–1.02)	0.43		
Sex: female								
Male	1.34 (0.98–1.83)	0.065			1.22 (0.93–1.59)	0.157		
Race: other								
Black	1.08 (0.53–2.22)	0.829			0.64 (0.35–1.18)	0.156		
White	0.94 (0.55–1.6)	0.814			0.73 (0.48–1.1)	0.131		
Smoke: No/Unknown								
YES	1.21 (0.88–1.66)	0.230	1.28 [0.91, 1.81]	0.158	1.14 (0.87–1.5)	0.347		
Ajcc_T: T1								
T2	1.67 (1.14–2.46)	0.009	1.49 [1.00, 2.22]	0.048	1.82 (1.31–2.53)	<0.001	1.66 [1.19, 2.33]	0.003
T3	2.63 (1.53–4.53)	<0.001	2.41 [1.39, 4.18]	0.002	2.94 (1.84–4.69)	<0.001	2.72 [1.70, 4.37]	<0.001
T4	4.27 (2.11–8.64)	<0.001	3.04 [1.44, 6.44]	0.004	2.91 (1.47–5.76)	0.002	2.09 [1.03, 4.24]	0.041
ajcc_N: N0								
N1	2.06 (1.43–2.95)	<0.001	2.00 [1.37, 2.92]	<0.001	1.88 (1.37–2.59)	<0.001	1.70 [1.22, 2.38]	0.002
N2+N3	2.42 (1.63–3.59)	<0.001	2.15 [1.42, 3.26]	<0.001	2.01 (1.42–2.85)	<0.001	1.73 [1.20, 2.49]	0.004
ajcc_M: M0								
M1	1.74 (0.96–3.17)	0.069			1.57 (0.92–2.68)	0.098		
MX	0.85 (0.58–1.24)	0.402			0.88 (0.64–1.21)	0.429		
stage: early stage								
Later stage	2.3 (1.66–3.19)	<0.001			1.88 (1.4–2.52)	<0.001		
Resection_site: Lower lobe								
Middle lobe	0.86 (0.34–2.15)	0.746			1.13 (0.56–2.26)	0.735		
Upper lobe	0.81 (0.58–1.12)	0.204			0.86 (0.64–1.15)	0.3		
Other site	1.66 (0.75–3.64)	0.209			2.33 (1.26–4.29)	0.007		
Radiotherapy: No/unknown								
Yes	1.86 (1.21–2.85)	0.004	1.41 [0.92, 2.17]	0.118	1.67 (1.16–2.4)	0.005	1.35 [0.94, 1.94]	0.106

The bold values emphasize the important variable.

### 3.2 Tumor genomic alterations between mutated and wild‐type patients

**FIGURE 3:**
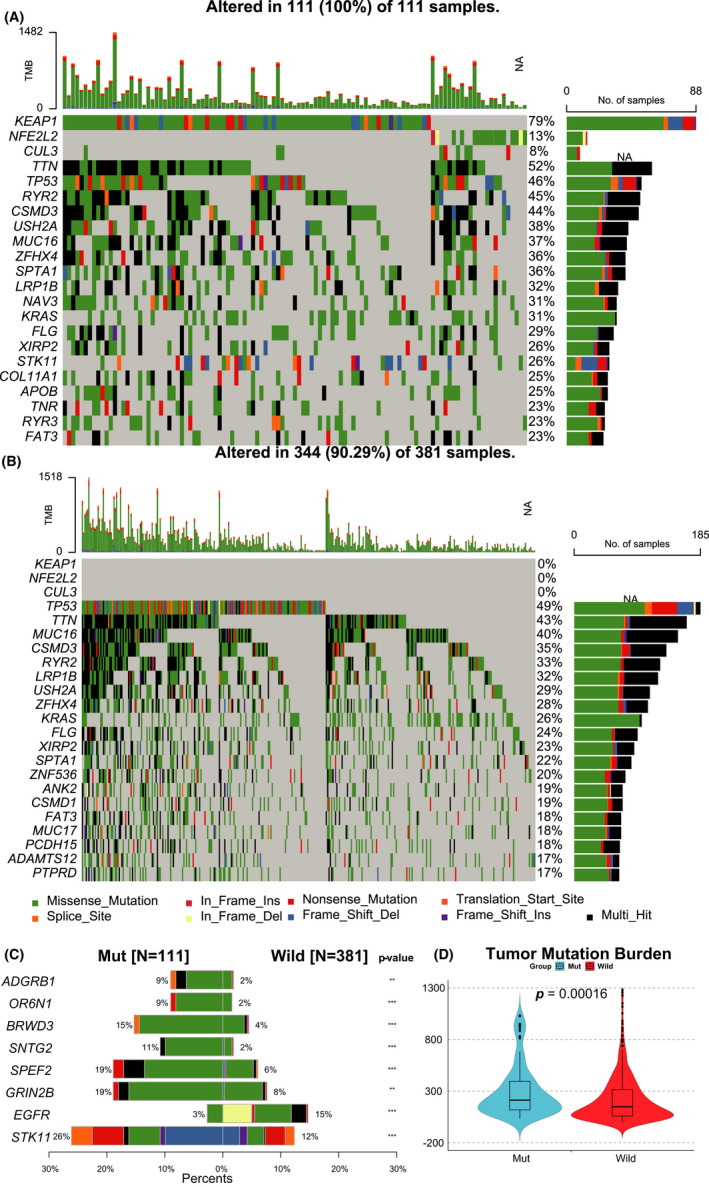
Genetic mutations related to Mut and Wild groups. Waterfall plot of (A) KEAP1/NFE2L2/CUL3 Mut and (B) Wild group summarizing the somatic alterations and copy number variations. Different colors annotated the type of alterations. (C) The top eight differential mutations in the Mut and the Wild groups and their distributions. *p* value indicated. (D) A violin plot is presenting the tumor mutation burden in the two groups. The differences between the two groups were compared through the Wilcoxon test. *p* values indicated

We performed the differential analysis in the somatic mutation distribution to the Mut and Wild group. Finally, 309 different somatic mutated genes between Mut and Wild groups were mined. The top 20 mutated genes’ distribution between the two groups was presented (Figure 3A,B), and KEAP1/NFE2L2/CUL3 genes were shown at the top. The most significant eight genes and their mutation frequency were shown in Figure 3C and Figure S3. Compared with the Wild group, the mutated frequency of Sperm Flagellar 2 (SPEF2: Mut, 19% vs. Wild, 6%, *p* < 0.001), Glutamate Ionotropic Receptor NMDA Type Subunit 2B (GRIN2B: 19% vs. 8%, *p* < 0.001), Syntrophin Gamma 2 (SNTG2: 11% vs. 2%, *p* < 0.001), Ryanodine Receptor 2 (RYR2: 45% vs. 33%, *p* < 0.001), and Serine/Threonine Kinase 11 (STK11: 26% vs. 12%, *p* < 0.001) were higher in Mut group, whereas epidermal growth factor receptor (EGFR: 3% vs. 15%, *p* < 0.001) was less frequently mutated in patients with KEAP1/NFE2L2/CUL3 mutations. The mutation rate of Tumor Protein P53 (TP53: 46% vs. 49%), Mucin 16, Cell Surface Associated (MUC16: 45% vs. 33%), and Titin (TTN: 52% vs. 43%) have no statistical differences. We mined that some important tumor drive genes differed significantly between the two groups, whereas others showed no differences. The Mut group's mutation rate was relatively higher than the Wild group, which can also be manifested at the tumor mutation burden level. The copy number variation data were integrated into the somatic mutations data to evaluate the tumor mutation burden precisely. The violin plot (Figure 3D) identified that the Mut group correlates with a higher tumor burden than the Wild group (*p* = 0.00016.). These data enabled us to comprehensively explore the KEAP1/NFE2L2/CUL3 mutated pathway's molecular characteristics.

### 3.3 DEGs and enrichment analysis

**FIGURE 4:**
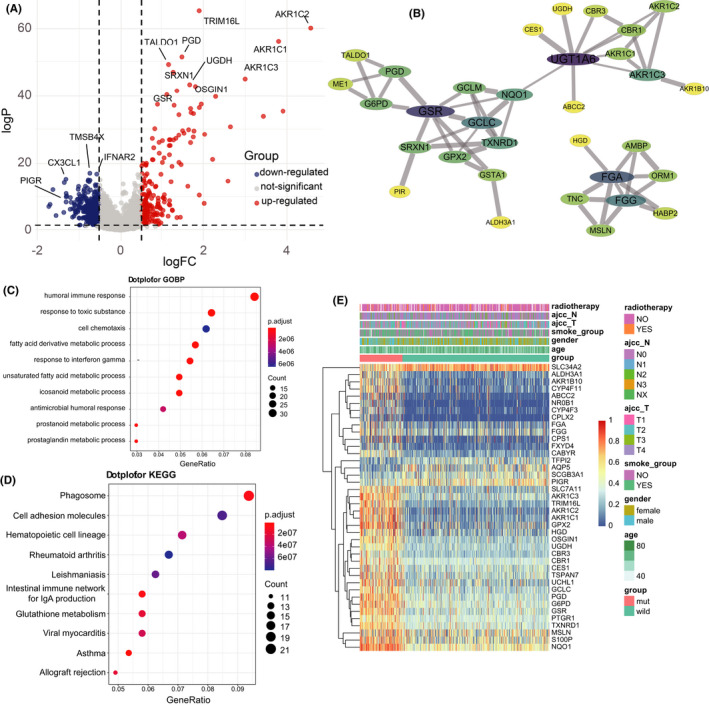
Differential and function enrichment analysis of DEmRNAs and ceRNA network. (A) Differential expressed genes between the KEAP1/NFE2L2/CUL3 Mut and the Wild groups were shown in a volcano plot. (B) Cytoscape's plugin “MCODE” found the hub genes in DEGs and established the PPI network. The node's size represents the degree of the gene, and the width of the line indicates the combined score. (C) Dot plot of GO‐BP and (D) KEGG pathway analysis to the DEGs (top 10). (E) Heatmap of the top 40 DEmRNAs and the phenotype of the two groups.

To identify the protein and other biologic characteristics between the Mut and the Wild groups. DEmRNAs (protein‐coding mRNAs) were detected by applying the limma package with the cutoff criteria (adjusted *p* value <0.05 and logFC >0.5). A total of 487 upregulated genes and 207 downregulated genes were detected in the Mut group. A volcano plot was also presented to show the differentially expressed genes ordered by the logFC value (Figure 4A). PPI network was constructed to identify the connections between the DE proteins (Figure 4B). We found that GSR (logFC = 1.36, *p* < 0.001), UGT1A6(logFC =1.24, *p* < 0.001), and the AKR Family proteins were the hub genes in the upregulated genes. Among the downregulated genes, PIGR (logFC = −1.72, *p* < 0.001) had the highest significant fold changes as shown in heatmap (Figure 4E).

On the other hand, GO and KEGG analyses were applied to the DEGs to exhibited enriched results (Figure 4C,D). The dot plot of GO enrichment analysis showed that the humoral immune response pathway was the most significant in the DEmRNAs of the Mut and Wild group. Other immune‐related pathways like positive regulation of cell activation, lymphocyte‐mediated immunity, and positive regulation of leukocyte activation have a suggestive effect on our following investigations. Concerning the KEGG’s results, apart from the immune‐associated pathways, phagocytosis, cell adhesion molecules, and glutathione metabolism have differed between the Mut and the Wild groups in KEGG pathways. Next, gene set enrichment analysis (GSEA) was conducted to attain better insight into the potential biologic procedures of functional effects that KEAP1/NFE2L2/CUL3 mutation connections within LUAD. As shown in Figure S4, the KEAP1/NFE2L2/CUL3 Mut group's ROS pathway oxidative phosphorylation and respiratory electron transport chain process were upregulated. In contrast, pathways such as NOTCH, KRAS, and JAK/STAT3 signaling were enriched in the Wild group. The GSEA results manifested that KEAP1/NFE2L2/CUL3 Mut group patients have a complex ROS mechanism that affects tumor development.

### 3.4 ceRNA network construction

**FIGURE 5:**
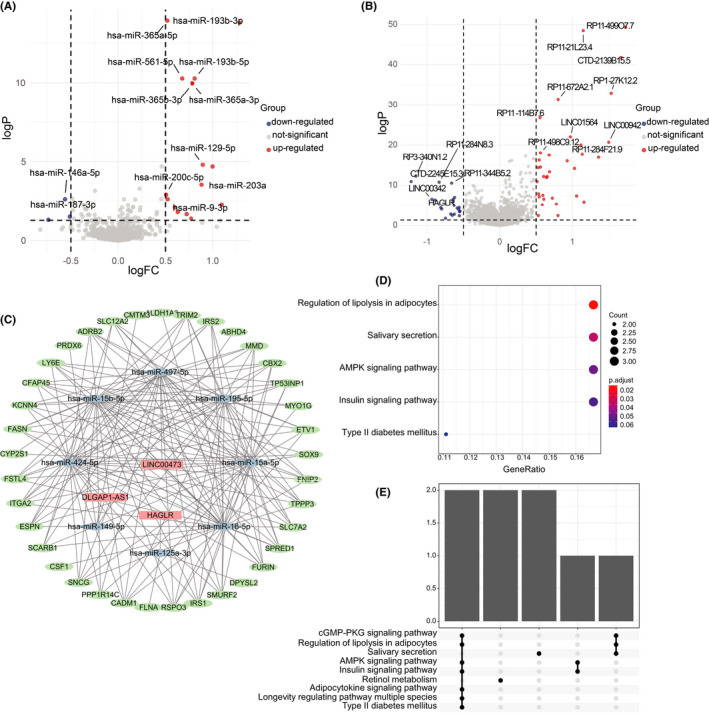
DEmiRNAs, DElncRNAs, and the ceRNA network analyses. (A) DEmiRNAs and (B) DElncRNAs between the Mut and Wild groups were presented in the volcano plots. (C) Dot plot of KEGG pathway analysis of the downstream mRNAs regulated by LINC00473 (D) Upset plot of hallmark enrichment analysis of the linc00473’s modified mRNAs. (E) The ceRNA network of the DElncRNA–DEmiRNA–DEmRNA demonstrates the cascade regulation relationship in the KEAP1/NFE2L2/CUL3 mutant LUAD patients

All expressed mature miRNAs were applied differential expression analysis to found the Mut and the Wild group's DEmiRNAs using the limma package (Figure 5A). Sixteen upregulated miRNAs and three downregulated miRNAs were identified statistically significant (*p* < 0.05, |logFC| > 0.5) (Figure 5B). We found that miR‐193b‐3p was the most significantly upregulated miRNA (logFC = 1.28, *p* < 0.001), As for the downregulated miRNAs, miR‐187‐3p was the most (logFC = −0.73, *p* < 0.001). Among all the 15,328 lncRNAs, there were 22 upregulated lncRNAs and 34 downregulated lncRNAs in the Mut group versus the Wild group (*p* < 0.05, |logFC| > 0.5). lncRNA RP11‐499O7.7(logFC = 1.73, *p* < 0.001) and lncRNA CTD‐2139B15.5(logFC = 1.66, *p* < 0.001) were the most significantly differentially expressed lncRNAs, both of them were upregulated in the Mut group. Given the ceRNA network mechanism's influence, a ceRNA network was established based on the above differential expression data to investigate the underlying association between lncRNAs, mRNAs, and protein‐coding mRNAs in LUAD. Finally, we identified three lncRNAs, eight miRNAs, and 36 mRNAs, and their interactions were predicted or validated in starBase, miRcode, and TargetScan databases. The network demonstrated the complex interactions through the visualization of the Cytoscape in Figure 5C. The key lncRNA in the ceRNA was LINC00473, which showed a significantly higher expression in KEAP1/NFE2L2/CUL3 Mut group (logFC = 0.72, *p* < 0.001). Moreover, our ceRNA network's downstream mRNAs were enriched in the AMPK signaling pathway, cGMP‐PKG signaling pathway, and other KEGG analysis pathways (Figure 5D,E).

### 3.5 Immune microenvironmental peculiarity

**FIGURE 6:**
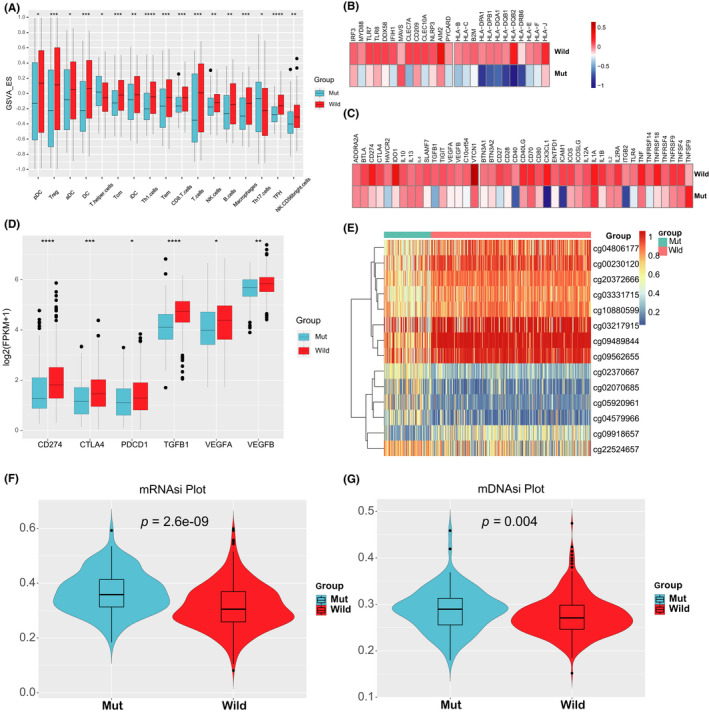
Immune landscape, methylation differences, and stemness indices of the KEAP1/NFE2L2/CUL3 Mut and Wild groups. (A) Comparison of each immune cell fraction between Mut and Wild groups, (B) relative expression level of molecules participated in innate immunity (left) and MHC‐I/II antigen‐presenting procedure (right), (C) relative expression level of immune coinhibitors (left) and costimulators (right), (D) the differential genes related to the immune checkpoint. *p < 0.05, **p < 0.01, ***p < 0.005, ****p < 0.001. (E) Heatmap of DMPs between the KEAP1/NFE2L2/CUL3 Mut and Wild groups (adjusted *p* value <0.05, |Deltabeta| >0.2); (F) mRNAsi and (G) mDNAsi differences of the two groups in the LUAD patients were displayed in the violin plots. *p* values indicated

The tumor microenvironment, where tumor cells proliferate, develop, and prepare for metastasis, is also infiltrated by immune cells and immune‐related molecules. Integral investigations to immune‐related genes, miRNAs, and other immune signatures were implemented to picture the thorough landscape of the KEAP1/NFE2L2/CUL3 mutant patients. First, we conducted a GSVA procedure and gained the Enrichment Score of 24 immune cell subsets. As shown in Figure 6A, the KEAP1/NFE2L2/CUL3 Mut group has a lower abundant level of immune cells than the Wild group. Only T helper and Th17 cells were upregulated in the Mut group. Then we estimated TIC (tumor‐infiltrating immune cells)’ proportions by CIBERSORT,^32^ ImmucellAI, EPIC, and QUANTISEQ, presenting similar results (Figure S5).

Since we have found significant differences in the composition of immune cells, the immune molecules were also analyzed on the basis of the expression profile. The differential analysis of the genes associated with the innate immunity and antigen‐presenting immune molecules between the KEAP1/NFE2L2/CUL3 Mut group and the Wild group were shown in Figure 6B, which demonstrated the same trends (all *p* < 0.05). Furthermore, 60 immune checkpoint genes, including 23 coinhibitors and 37 costimulators, were compared between the KEAP1/NFE2L2/CUL3 Mut and Wild groups. Only significant (*p* < 0.05) and concordant results were discussed. The large majority of immune checkpoint genes, as shown in the heatmap (Figure 6C), were observed expressed higher in the Wild group (most *p* < 0.05, such as PD‐L1 [CD274], CTLA4, PD‐1 [PDCD1], TGFB1, VEGFA, and VEGFB, Figure 6D). Next, we analyzed other immune‐related genes expression between the two groups.^33^ Among the top 10 differential immune genes (Figure S5), five immune genes were related to antimicrobials functions, four cytokines and cytokine receptor‐related genes were downregulated in the Wild group. Furthermore, CX3CL1(C‐X3‐C Motif Chemokine Ligand 1) was a chemokine that significantly upregulated in the Wild group. These findings indicated a connection between the cytokine or chemokine's function and microenvironment changes of the KEAP1/NFE2L2/CUL3 Mut group.

### 3.6 Tumor stemness differences

Malta's study^28^ demonstrated that Tumor stemness could be accounted for RNA stemness index (RNAsi) and DNA stemness index (DNAsi) which were based on mRNA expression and DNA methylation by using the one‐class logistic regression (OCLR) machine‐learning algorithm. In our study, we applied the Chip Analysis Methylation Pipeline (ChAMP) R package to mining the differential methylated positions (DMPs) and the differential methylation regions (DMRs). As shown in Figure 6E, the top 14 differential DMPs (adjusted *p* value <0.05, |Deltabeta| >0.2) between the KEAP1/NFE2L2/CUL3 Mut and Wild groups were presented with a heatmap (Figure S6A). The most differentially methylated position (DMP), cg10880599 on chromosome 14, was hypermethylated in the Wild group (Figure S6E), which might downregulate the expression of gene GPX2. We also found significant CpGs enriched some other DEGs (Figure S6B,C).

The GSEA analysis of the DMPs and DMRs identified that some pathways about cancers and glutamate metabolism were enriched in coinciding with the DEmRNAs’ result. The differences in DNA methylation were validated in the DNAsi. In Figure 6F,G, we compared the DNA stemness index and RNA stemness index. The Mut group had a significantly higher value of both indices (493 patients, 111 KEAP1/NFE2L2/CUL3 Mut and 382 Wild groups, RNAsi: *p* < 0.001; DNAsi: *p* = 0.004). These results fitted the initial investigations that the Mut group patients might have more oncogenic dedifferentiation.

### 3.7 Validation of the expression levels of eight hub factors in LUAD tumor tissues

**FIGURE 7:**
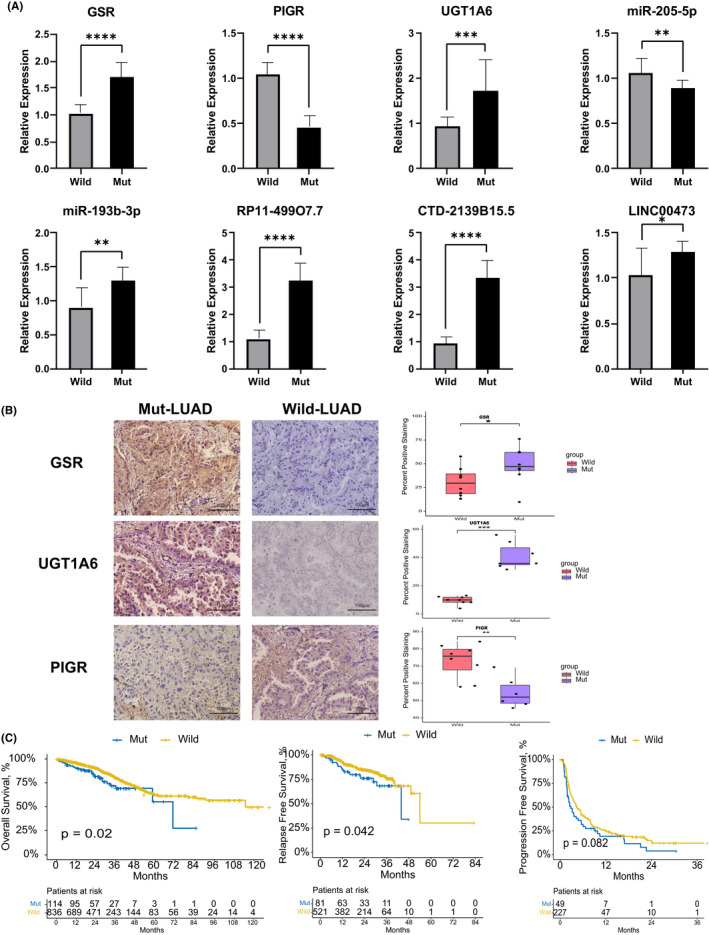
Validation of expression levels in our own cohort and the external validation of survival analysis. (A) The expression level of GSR, PIGR, UGT1A6, miR‐205‐5p, miR‐193b‐3p, RP11‐499O7.7, CTD‐2139B15.5, and LINC00473. *p < 0.05, **p < 0.01, ***p < 0.005, ****p < 0.001. (B) The IHC staining of three DEGs in Mut and Wild KEAP1 LUAD tissue samples. Quantification of percent positive regions (right) was performed using the IHC profiler plugin for ImageJ. Data are presented as the mean ± SD. (C) Kaplan–Meier survival curves between the Mut and the Wild groups in OS, RFS, and PFS

To better estimated the above bioinformatics results obtained from the public databases, we collected 50 LUAD samples and tested their mutation status using Sanger's sequencing. Table S2 showed that 10 missense KEAP1 mutations, including two deletions, were detected in seven patients (16%), whereas only one NFE2L2 mutation was detected in one patient (2%). Next, we selected these eight KEAP1/NFE2L2 Mut LUAD samples and paired 16 Wild LUAD samples to test the expression levels of eight significant differential hub genes, miRNAs, and lncRNAs was fundamental in the ceRNA network. As shown in Figure 7A, the quantitative rt‐qPCR array showed enhanced expression in upregulated factors such as GSR and UGT1A6. Alternatively, the expression of PIGR and miR‐205‐5p has significantly diminished in the Wild group patients. The results were generally compatible with the previous differential analysis. Additionally, the IHC analysis with paraffin continuous tissue sections on the three differential genes verified that GSR and UGT1A6 have significantly higher expression in the Mut patient group than the Wild group, and the Wild LUAD patient had a stronger PIGR expression. These important findings further emphasize that the differentiating factors we figured out in silicon analysis are biologically meaningful.

### 3.8 The survival analysis of external validation cohort

The external validation cohort was browsed and downloaded from cBioportal, which was combined from the studies such as MSK, MSKCC, and OncoSG and provided the mutated status. OS and RFS between the KEAP1/NFE2L2/CUL3 Mut and Wild groups were considerably different. As shown in Figure 7C, PFS’s difference was not statistically significant, but it had a low *P* value as well. Inconsistent with the TCGA database, LUAD patients with KEAP1/NFE2L2/CUL3 mutations have a worse prognosis in terms of survival and disease progression. The cross‐validation of internal and external strengthened the clinical value of our research. Combined with Jessica's study,^34^ the KEAP1/NFE2L2/CUL3 pathway alterations may play a pivotal role in carcinogenesis, invasion, and treatment resistance.

## 4 DISCUSSION

KEAP1/NFE2L2/CUL3 alterations in LUAD jeopardized the normal function of the antioxidant signaling pathway,^35^, ^36^ which contributed to the tumorigenesis and resistance to target treatments or chemotherapies in the patients.^37^ Nevertheless, the therapeutic drugs targeting the KEAP1/NFE2L2/CUL3 pathway mutations were still underdeveloped. The independent prognostic value of the three gene mutations was validated in the present study. The multi‐omics genetic analysis and tumor immune microenvironment characterization revealed the latent mechanism and developed our understanding, contributing to discovering new therapeutic target drugs.

The NFE2L2 pathway was mainly comprised of cullin 3 (CUL3)/kelch‐like ECH‐associated protein 1 (KEAP1) and nuclear factor erythroid 2‐like 2 (NFE2L2). It was well known that KEAP1 functions as an adaptor for CUL3‐based E3 ligase to regulate proteasomal degradation of NFE2L2,^38^ the KEAP1/NFE2L2/CUL3 mutations caused the abnormal activation of the NFE2L2 pathway, which drive cancer progression.^39^ Recent studies have reported that KEAP1/NFE2L2/CUL3 mutated in many cancers and led to worse survival outcomes in many cancers,^35^, ^38^, ^40^, ^41^, ^42^, ^43^, ^44^, ^45^ our survival analysis also confirmed this. The alteration rate in lung adenocarcinoma was over 20%. Frank et al. reported that KEAP1 mutations spread over the whole protein while the NFE2L2 is often mutated in specific hotspot regions. In the study of Goeman et al., variations of KEAP1/NFE2L2/CUL3 in LUAD were defined as a molecular subtype rapidly progressing.^46^ Many studies revealed that the NFE2L2 pathway was a “double‐edged sword” in cancer.^47^ It could resist oxidative damage from the external environment, thus preventing the carcinogenesis of normal cells.^48^ For example, the full function NEF2L2 genotype could protect the smoker against the oxidant and chemical stress which could be carcinogenic.^49^ However, emerging evidence have illustrated that NFE2L2’ hyperactivation promotes metabolic reprogramming via redirecting glucose and glutamine to anabolic pathways.^50^, ^51^ Su et al. revealed that NFE2L2 activated micropinocytosis in pancreatic ductal adenocarcinoma for the energy supplies to tumor cells autophagy‐deficient.^52^ Whereas connections with the antioxidant response, metabolic reprogramming, and autophagy have been demonstrated, definitive mechanisms underlying the NFE2L2 pathway remain highly sought after.

The treatment of lung cancer was gradually developing, but the patients with KEAP1/NFE2L2/CUL3 mutations were in a dilemma. On the one hand, these patients were not eligible for targeting treatments because of the lack of activating genetic mutations or fusions and the resistance to the kinase inhibitor drugs,^53^ on the other hand, previous studies had demonstrated that the genetic alterations on the NFE2L2 pathway's gene would result in tumor resistances against chemotherapeutic agents in NSCLC.^37^, ^54^, ^55^ Consequently, the optimal choice for the KEAP1/NFE2L2/CUL3 mutant patients was immunotherapy compared with other treatments.^56^, ^57^, ^58^, ^59^

Our results show that LUAD patients with EGFR mutations often do not have KEAP1/NFE2L2/CUL3 mutations simultaneously, called the mutually exclusive pattern SNPs.^60^, ^61^ However, in Hellyer’ study, 7% (17 in 228) of EGFR‐mutant NSCLC patients also carried alterations in KEAP1/NFE2L2/CUL3, the patients with the comutation of KEAP1/NFE2L2/CUL3 had a shorter median time to treatment resistance on EGFR TKI (4.7 months) than the wild‐type matched cohort (13.0 months).^34^ Besides, we found that STK11, SPEF2, and other gene mutations were significantly higher in patients with NFE2L2 pathway mutations than in the Wild group. The STK11 (or LKB1) gene is a tumor suppressor gene. Its mutation often resulted in tumor metastasis and poor survival in NCSLC.^57^, ^62^ The relationship between STK11 and KEAP1 mutations in LUAD is worth further investigation. Our differential analysis on the mRNA level showed that GRS, UGT1A6, were upregulated in the Mut group, and they were all downstream genes of the KEAP1/NFE2L2/CUL3 pathway. Glutathione S‐Reductase(GRS) gene, encoding glutathione (GSH) reductase, had a crucial role in the cancer progression and treatment response via the metabolic of glutamine in TME, and Baity et al. found that GSR copy number loss is common in LUAD, which might be a biomarker for personalized therapy in the future.^63^, ^64^ UDP Glucuronosyltransferase Family 1 Member A6 (UGT1A6) was related to the lipid metabolism by transforming small lipophilic molecules into hydrophilic molecules, and Li et al. found that its overexpression in LUAD has relationship with a worse prognosis.^65^, ^66^ Kua et al. also reported that the UGT1A6 polymorphisms might modulate lung cancer risk. The expression of polymeric immunoglobulin receptor (PIGR) in the Mut group was downregulated. The loss of pIgR expression is associated with cell proliferation and poor prognosis in lung cancer.^67^ However, in Ai et al.’ study,^68^ its high expression also was identified as a role between induction of epithelial–mesenchymal transition (EMT) and hepatocellular carcinoma (HCC) metastasis. The two sides of immune defense and immune betrayal of pIgR in LUAD need further exploration. However, the associations between KEAP1/NFE2L2/CUL3 mutations and the expression of these genes in LUAD have not been reported yet.

With regard to the DEmiRNAs and DElncRNAs, in our study, the overexpression of hsa‐miR‐193b‐3p in the Mut was evident. The previous study reported its importance in the progression of gastric cancer and colon cancer.^69^, ^70^ Although it was considered to have tumor suppressor functions in acute myeloid leukemia,^71^ the recent study of Zhang found that miR‐193b‐3p was upregulated in NSCLC, which verified the miR‐193‐193b‐3p could serve as a biomarker of NSCLC,^72^ our findings further revealed that the overexpression of miR‐193b‐3p might have relationships with the KEAP1/NFE2L2/CUL3 mutations. As to the most significant downregulated miRNA, miR‐187‐3p played a vital role in tumor inhibition and chemoresistance rescuers in NSCLC.^73^, ^74^ The DElncRNA RP11‐499O7.7 and CTD‐2139B15.5 were the first time to report in the present study that the overexpression of the KEAP1/NFE2L2/CUL3 mutant LUAD patients, the functions, and mechanism requires further explorations. Thus, we constructed the ceRNA network and identified the crucial lncRNA LINC00473. lncRNA LINC00473 is located on the human chromosome 6p27 and has been overexpressed in various malignant tumors including LUAD.^75^, ^76^, ^77^, ^78^, ^79^, ^80^ Our findings were consistent with the previous study and implied that LINC00473 might be a novel driver of lncRNA in tumor progression and an extensive anticancer therapeutic target. The downstream genes in ceRNA were enriched in some vital pathways associated with tumor progression. FNIP2, as the highest combined score node in ceRNA, was identified to play an important role in kidney tumor suppression, whereas its function in LUAD remains unclear.

The critical role of the tumor environment (TME) in LUAD has been elucidated in various studies.^81^, ^82^ We investigated the infiltration of the immune cells via GSVA analysis. The present study demonstrated that the KEAP1/NFE2L2/CUL3 mutations might be correlated to the lower immune infiltration and higher tumor mutation burden. The expression of MHC class II in the Mut group was markedly decreased. As we know, one of the immunoevasion mechanisms is that the cancer cells hide their tumor antigens. Johnson's study illustrated that the inadequate MHCII expression in LUAD resulted in a lower response to immunotherapy.^83^ We also found a markable difference in the expression of the PD‐L1 (CD274), PDCD1, and CTLA4 between the two groups. These investigations about immune infiltration and tumor environment indicated that the Wild group patients might have a higher immunotherapy response rate due to the TME status and the KEAP1/NFE2L2/CUL3 alterations that contribute to the tumor immune escape and “cold” tumor's formation.^7^ Given that the immunotherapy’ response rate was still low at 14–20% in unselected patients, and the recent study revealed the uptake of glutamine and lipids was controlled by tumor cells, suggesting that targeting glutamine metabolism could be used as a specific strategy to inhibit tumor growth and change the immunophenotype of TME.^84^

Our research still has certain limitations. Since KEAP1/NFE2L2/CUL3 gene mutations have not received enough attention in the clinical practice of lung adenocarcinoma, these three genes are not included in routine postoperative pathologic sample gene mutation detection in our hospital. Our cohort for the validation needs more appropriate patients included. Therefore, our further research needs more samples to provide a more accurate subgroup analysis of KEAP1/NFE2L2/CUL3 pathway mutations so as to exhibit a deeper insight into KEAP1/NFE2L2/CUL3 pathway mutation in lung adenocarcinoma progression. The current study is a preliminary validation of the key difference factors. More research needs to be done to figure out the underlying molecular mechanism of mutations of the KEAP1/NFE2L2/CUL3 pathway in lung adenocarcinoma, and there is still a long way to go to target these mutations as a new therapeutic strategy.

Generally, our study comprehensively analyzed the multiplatform data of TCGA to compare the biologic characteristics, intrinsic heterogeneities, and clinical features of the KEAP1/NFE2L2/CUL3 mutant and wild lung adenocarcinoma patients. It is imperative to mine the underlying mechanisms and characteristics of KEAP1/NFE2L2/CUL3 mutations in lung adenocarcinoma and accelerate the investigations of the pathway and the targeted drugs.

## ETHICS APPROVAL AND CONSENT TO PARTICIPANTS

Our study was approved by the Ethics Committee on Human Research of the Zhongshan Hospital, Fudan University. All the informed consent has been obtained from each participant. Other data from the public database did not require ethical consent.

## CONSENT FOR PUBLICATION

Not applicable.

## CONFLICT OF INTEREST

The authors declare no competing interests in this work.

## AUTHOR CONTRIBUTIONS

Songtao Xu and Yulei Qiao conceived the study. Xing Jin, Yuansheng Zheng, and Zhencong Chen performed most of the bioinformatics analysis and wrote the manuscript. Fei Wang, Guoshu Bi, Ming Li, Jiaqi Liang, and Qihai Sui analyzed the data. Yunyi Bian and Zhengyang Hu helped design and manuscript editing.

## Supporting information

Fig S1

Fig S2

Fig S3

Fig S4

Fig S5

Fig S6

Table S1

Table S2

## Data Availability

Data adopted in this study are available in TCGA (http://portal.gdc.cancer.gov/) and UCSC Xena Browser (http://xena.ucsc.edu/). The main R code scripts file were uploaded to Github (https://github.com/xjin15/KEAP1‐NFE2L2‐CUL3‐analysis‐code/tree/main).
