# Supplementary figures and images for: Integrated analysis of patients with KEAP1/NFE2L2/CUL3 mutations in lung adenocarcinomas
Source: Cancer Med. 2021 Oct 6;10(23):8673–92. doi: 10.1002/cam4.4338 (PMC8633244; doi:10.1002/cam4.4338)

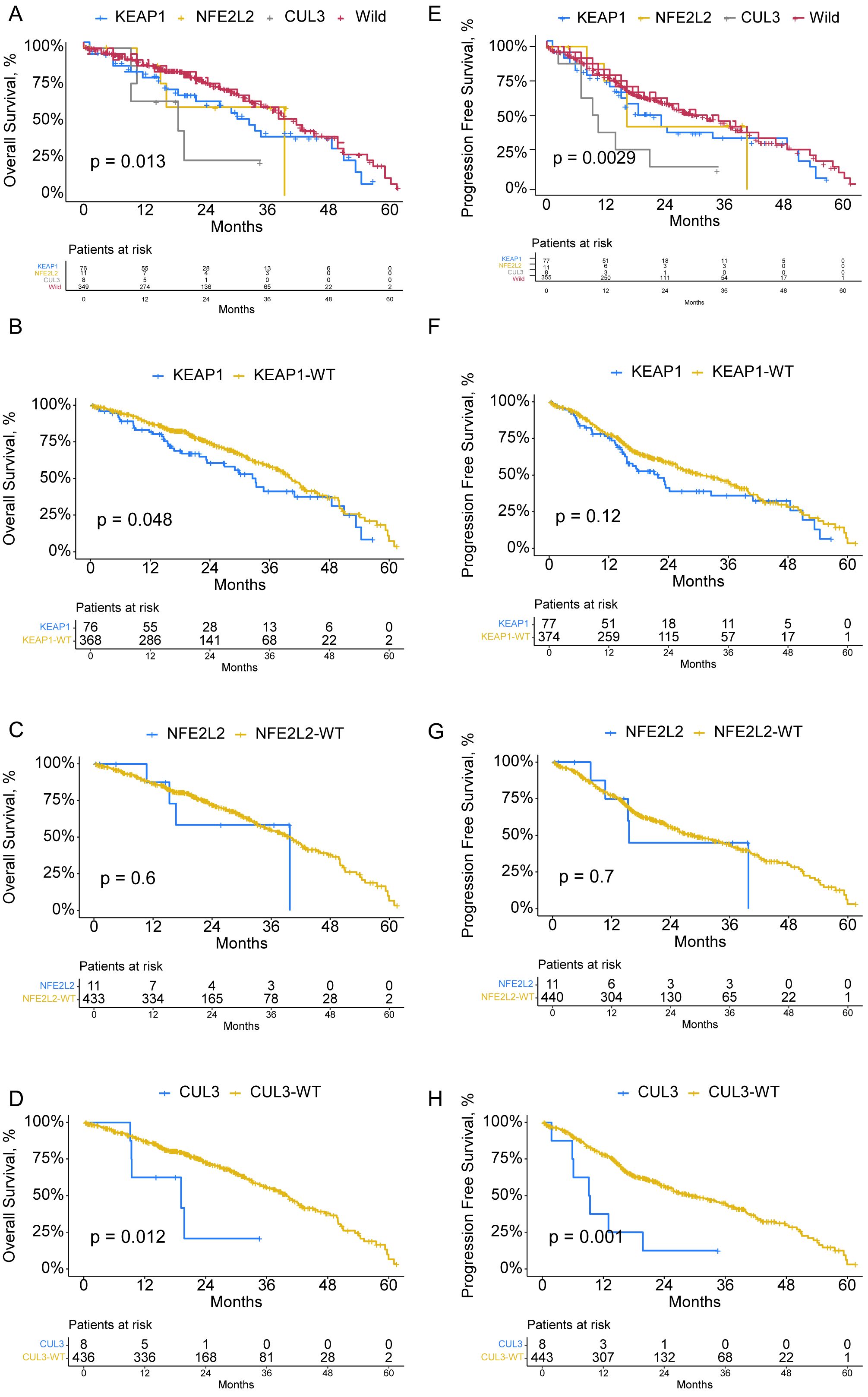

Supplement: Supplementary file 1 — Fig S1 [file CAM4-10-8673-s006.jpg]

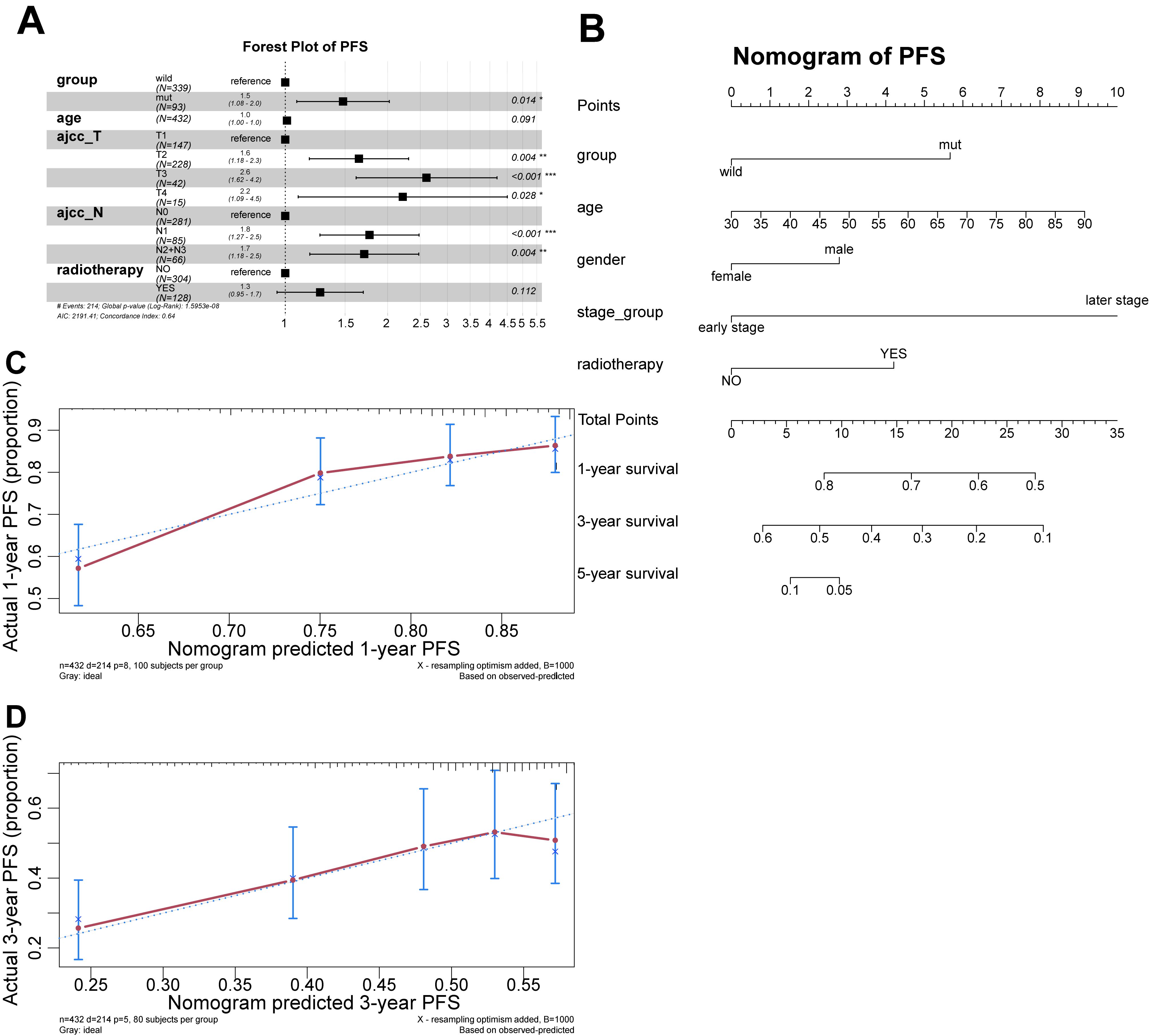

Supplement: Supplementary file 2 — Fig S2 [file CAM4-10-8673-s001.jpg]

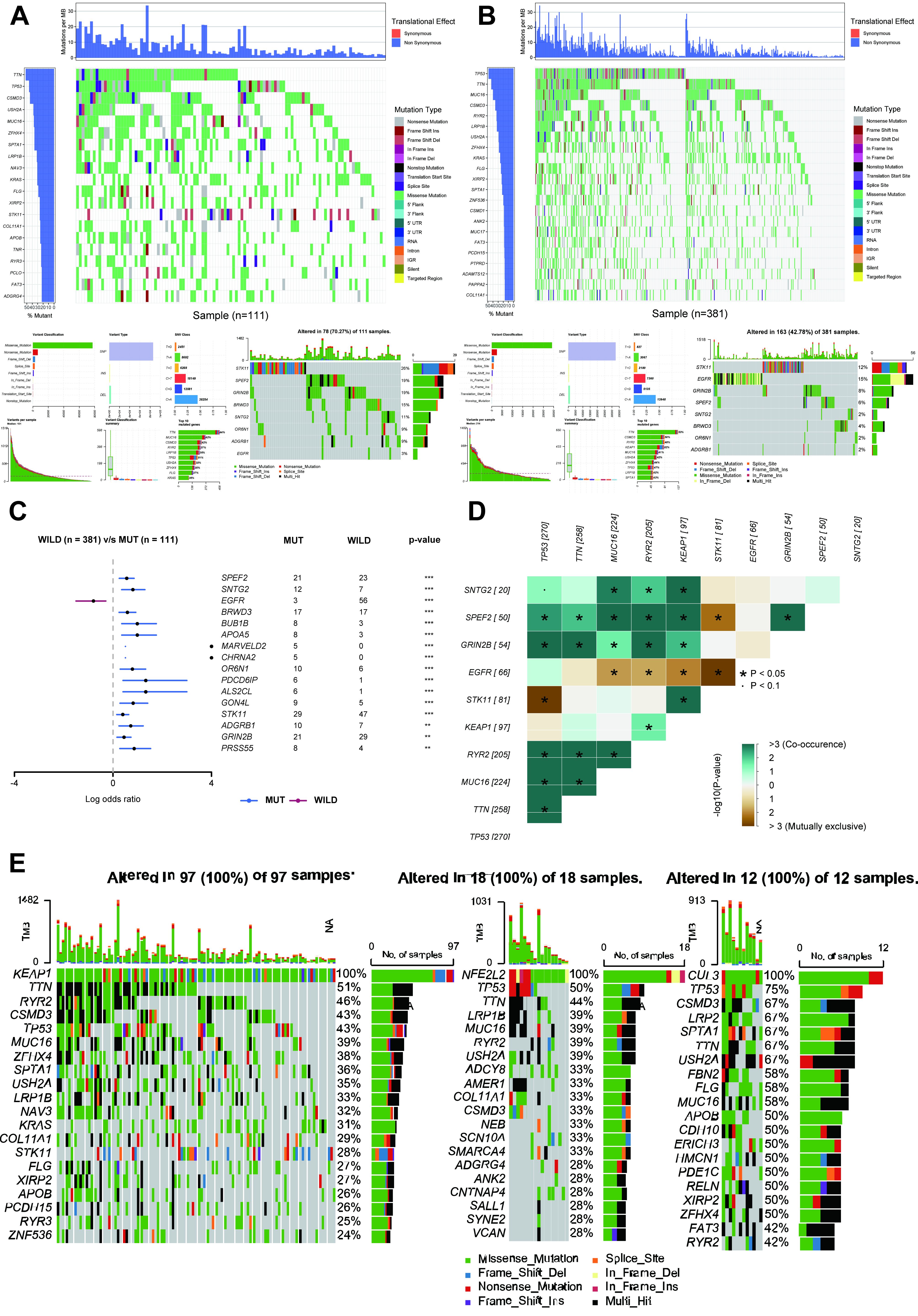

Supplement: Supplementary file 3 — Fig S3 [file CAM4-10-8673-s004.jpg]

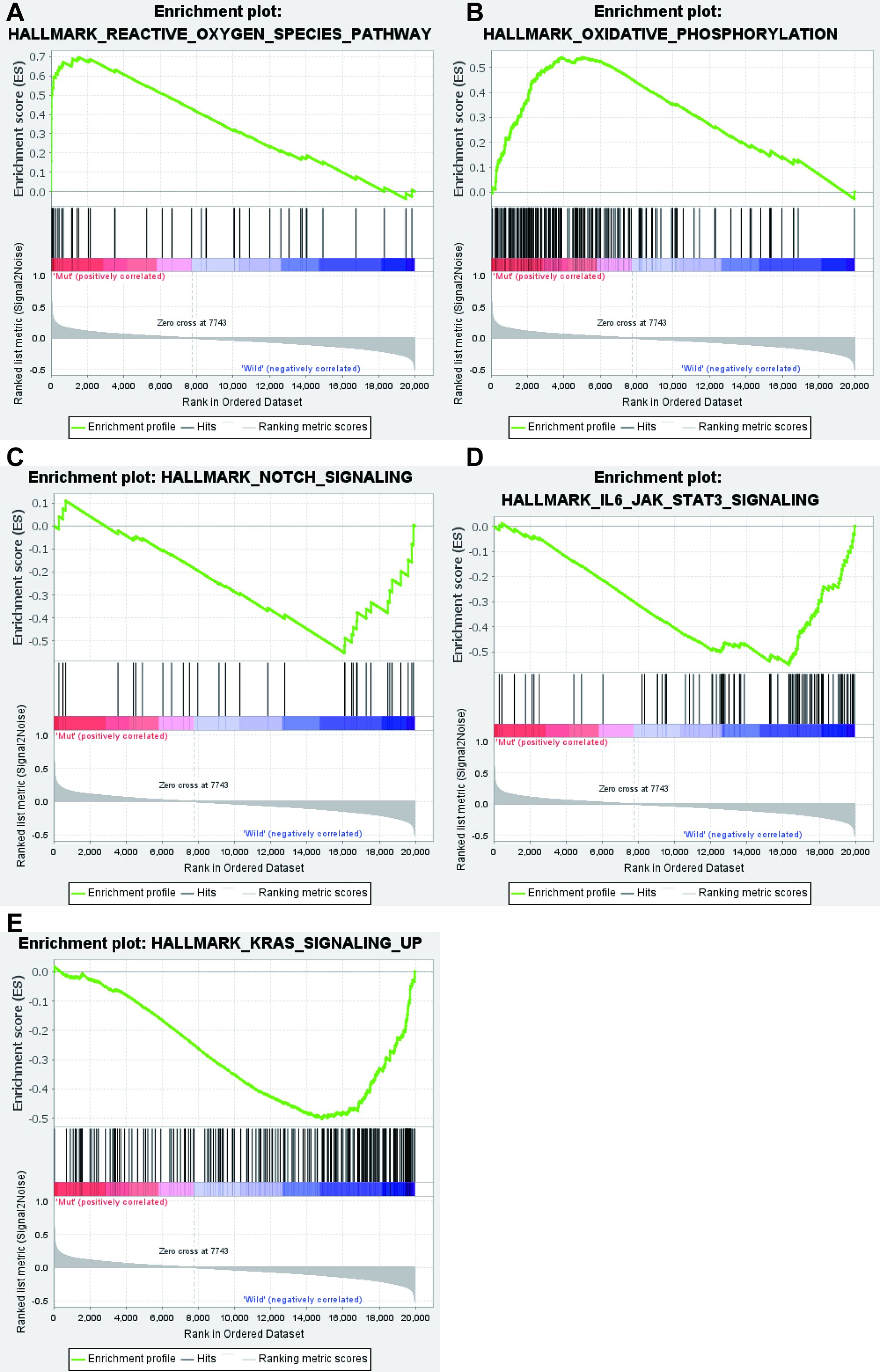

Supplement: Supplementary file 4 — Fig S4 [file CAM4-10-8673-s002.jpg]

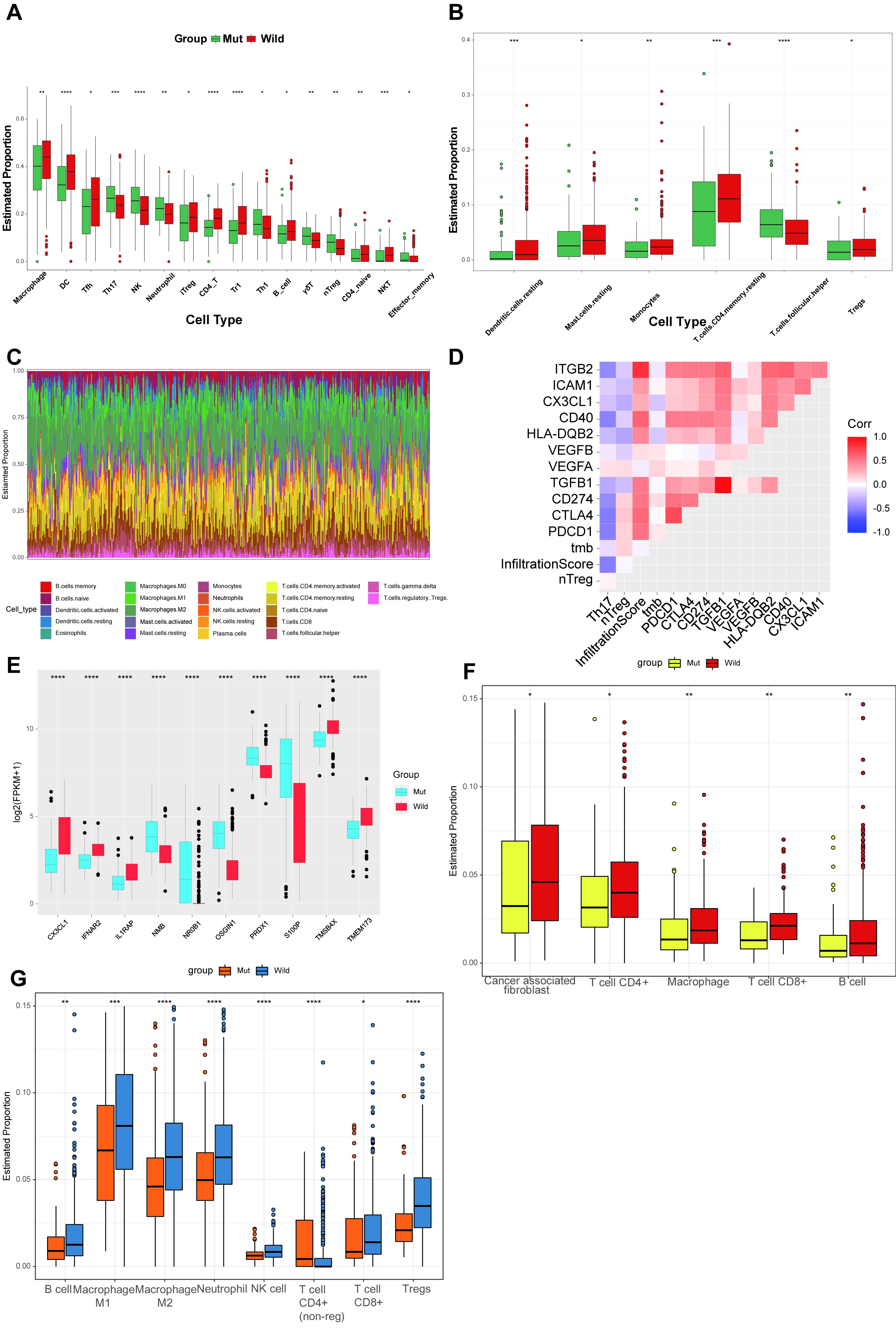

Supplement: Supplementary file 5 — Fig S5 [file CAM4-10-8673-s008.jpg]

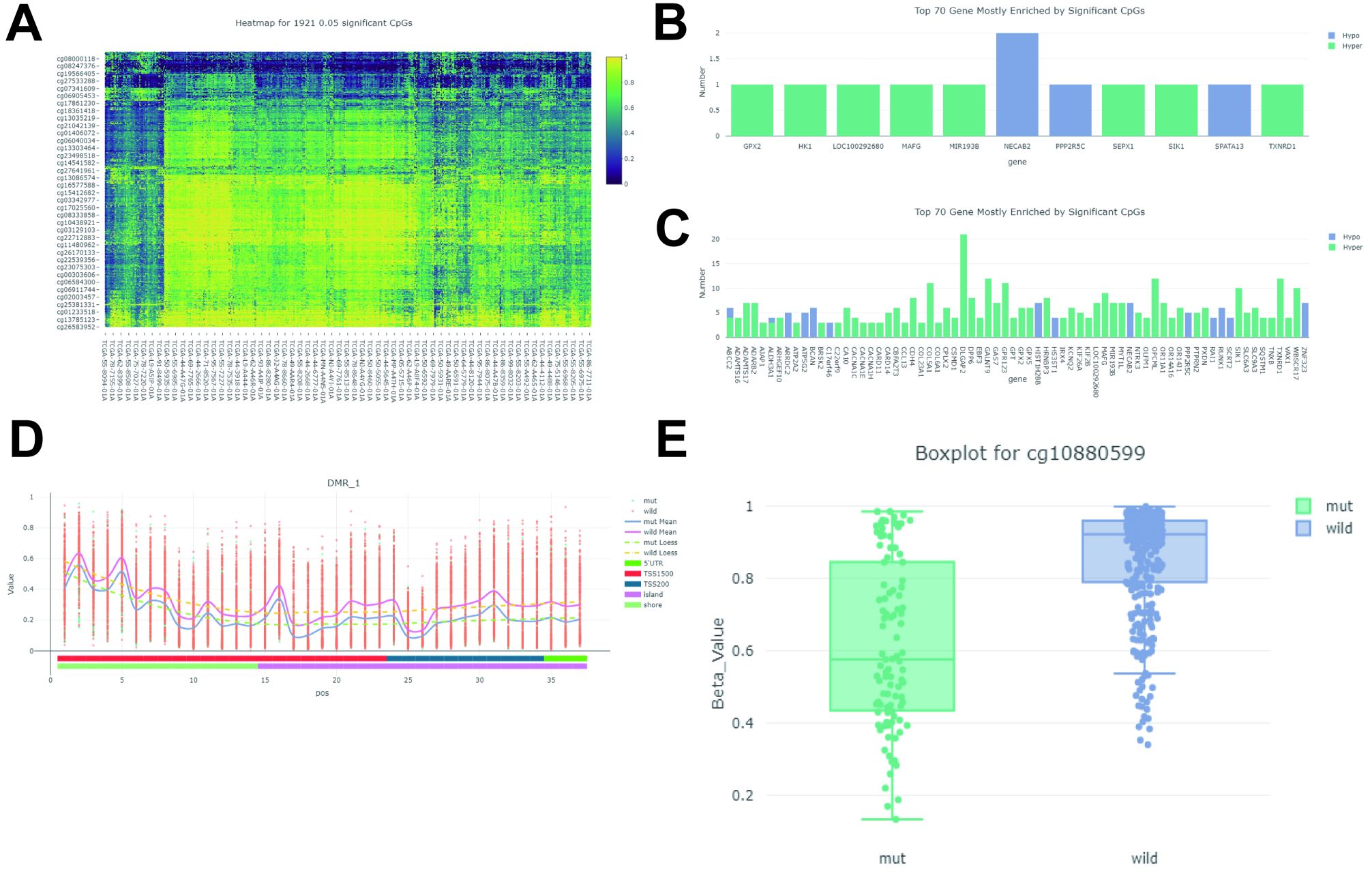

Supplement: Supplementary file 6 — Fig S6 [file CAM4-10-8673-s003.jpg]

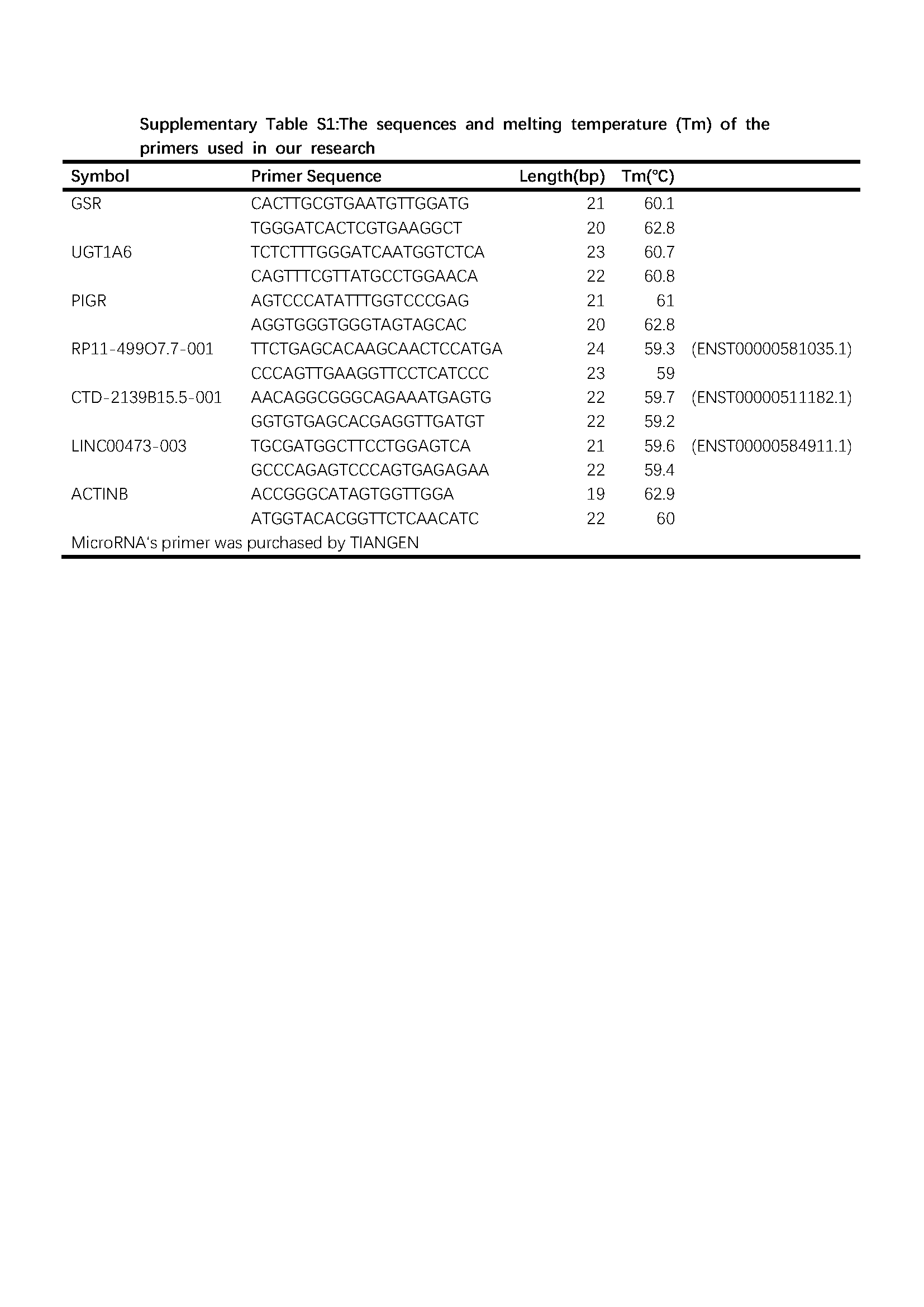

Supplement: Supplementary file 7 — Table S1 [file CAM4-10-8673-s005.tiff]

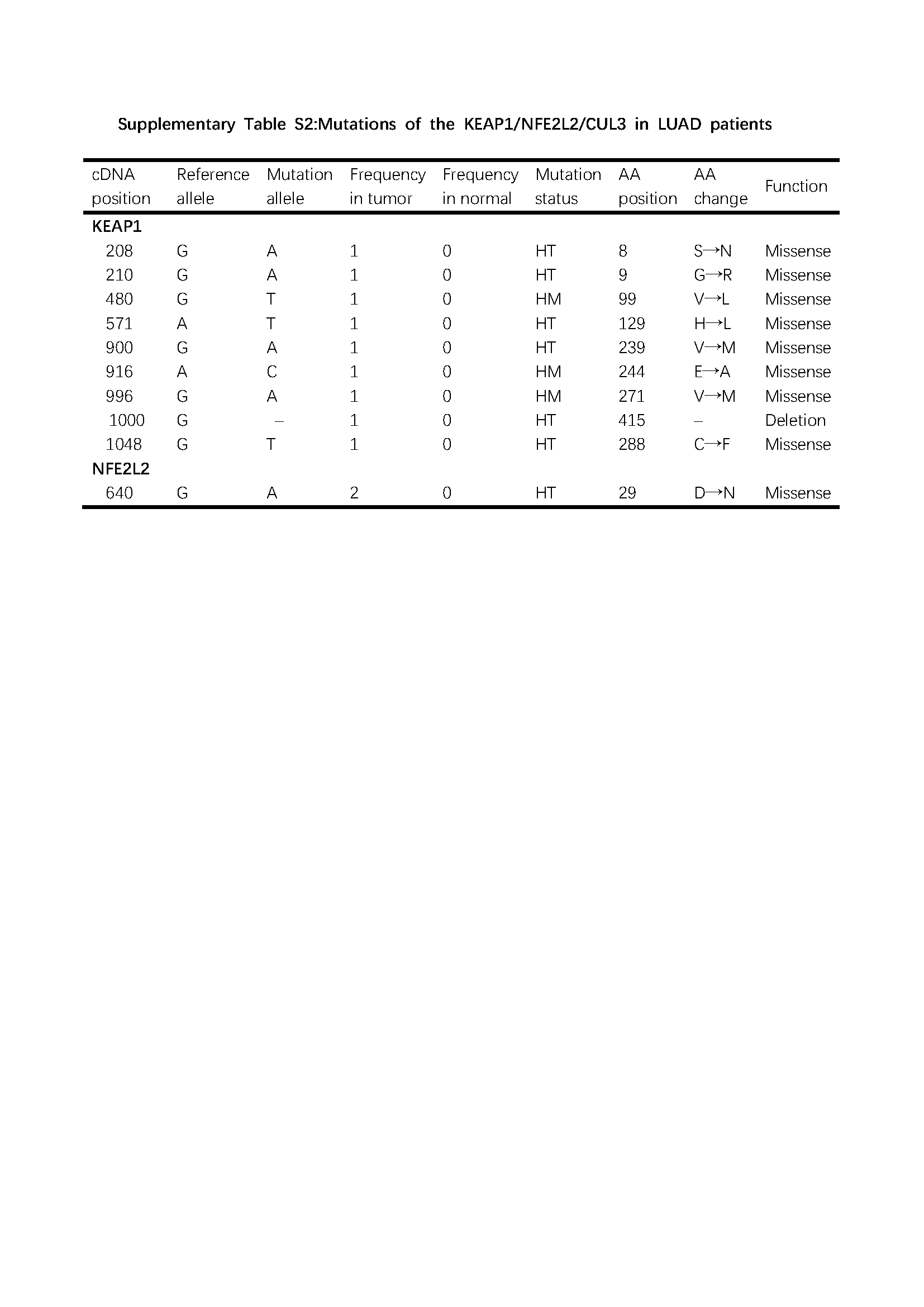

Supplement: Supplementary file 8 — Table S2 [file CAM4-10-8673-s007.tiff]
